# Editor-in-Chief’s welcome to *Hepatology, Medicine and Policy*

**DOI:** 10.1186/s41124-016-0005-9

**Published:** 2016-04-13

**Authors:** Jeffrey V. Lazarus

**Affiliations:** CHIP, Centre for Health and Infectious Disease Research and WHO Collaborating Centre on HIV and Viral Hepatitis, Rigshospitalet, University of Copenhagen, Copenhagen, Denmark

## Abstract

*Hepatology, Medicine and Policy* (*HMAP*), a new open-access peer-reviewed journal, is making its debut at a time when the hepatitis field is seeing great progress but still has far to go. The World Health Organization and many countries have shown increasing interest in viral hepatitis in recent years, helping to foster a stronger response to this group of diseases. Meanwhile, alcohol-associated cirrhosis and alcohol-associated liver cancer continue to take a heavy toll worldwide, as does non-alcoholic fatty liver disease. The lack of a unified strategic response to viral hepatitis and other liver diseases is the impetus for launching *HMAP*, which will publish policy, public health and social science articles alongside clinical science articles. It will encourage submissions in diverse domains such as disease prevention and management, epidemiology, economics, health behavior, health service delivery, ethics, human rights, and the role of laws, policies and clinical guidelines in shaping health initiatives. The current attention to powerful new hepatitis C treatments presents a strategic opportunity to more comprehensively address the full constellation of biomedical and social issues relating to liver health. *HMAP* is committed to publishing research and policy articles that help to drive forward this broader agenda.

## Who needs a magnifying lens?

Pens? Tote bags? T-shirts? I still remember the lengthy discussion. It was 2006, and I had been tapped as the World Health Organization (WHO) European office’s staff representative for the planning of “World Hepatitis Awareness Day,” as it was called back then.

The newly formed European Liver Patients Association (ELPA) was determined to raise the profile of this little-known event, and had engaged WHO/Europe, the European Association for the Study of the Liver (EASL) and other organizations in an ambitious publicity campaign.

We finally decided that a key chain displaying the World Hepatitis Awareness Day logo would be a suitable “giveaway” item. And we ordered a bulk supply of a flat wallet-sized magnifying lens, also marked with the logo. This additional item was for advocates who wanted to protest public officials’ lack of attention to hepatitis—the idea was for them to dramatically brandish their lenses at the appropriate moment and say that they were searching unsuccessfully for some acknowledgement of hepatitis. Such was the state of hepatitis awareness at the time.

I would like to say we took the world by storm. But in truth, we didn’t even take my office by storm, and the European media were largely indifferent to our efforts.

Despite the lackluster response, I was quite pleased with one outcome that might be best characterized as “unintentional stealth advocacy from within.” I had harbored some doubts about the little magnifying lenses, but this investment demonstrated its worth when I saw a WHO regional deputy director using one several months later. She said that she kept it for reading fine print!

Ten years on from this somewhat ragtag effort, my continuing engagement in the global response to liver diseases has led me to found *Hepatology, Medicine and Policy* (*HMAP*). An open-access peer-reviewed journal, *HMAP* is making its debut at the time of the World Health Assembly’s approval of the first Global Health Sector Strategy on Viral Hepatitis [1]. The establishment of *HMAP* compels me to reflect on both how far we have come, in terms of global efforts to combat hepatitis and other liver diseases, and how far we still have to go.

## A half-full or half-empty glass?

On the one hand, the World Health Assembly’s first-ever viral hepatitis resolution in 2010 [2] spurred WHO to step up and provide much-needed leadership and technical guidance. Far more people both within and outside of public health circles are now aware of the magnitude of hepatitis epidemics in many countries, and some governments have taken important steps toward developing effective responses [3]. The patient-led World Hepatitis Alliance—with which *HMAP* is proudly affiliated—has a thriving constituency comprised of 230 member organizations in 81 countries [4], and is a strong catalyst for policy change and civil society involvement globally.

Then there is the incredibly exciting news in the realm of hepatitis C treatment, with the latest drugs achieving such high cure rates that some stakeholders think it is time to set our sights on the worldwide elimination of this disease [1, 5, 6].

On the other hand, there is still no cure for hepatitis B and no vaccine against hepatitis C. Efforts to reduce illness and death from both diseases are hampered by the fact that many of those carrying both viruses are unaware of their infection [1]. Furthermore, the cost of treatment—especially treatment with the newest hepatitis C drugs—presents a major obstacle to effective clinical care in many countries.

Hepatitis A annually strikes an estimated 1.4 million people [7], and hepatitis E, an estimated 20 million people [8], with household and community poverty often serving as underlying factors in the transmission of these viruses through contaminated water. Although the burden of disease from both viruses is concentrated in developing countries, evidence is emerging to suggest that the zoonatic transmission of hepatitis E virus in developed countries—primarily through the consumption of meat from infected animals such as pigs, as well as through the transfusion of contaminated blood products—may be a greater cause of illness than previously recognized, and there are important unanswered questions about the epidemiology of hepatitis E disease [9, 10].

## … And what of the other glasses?

While hepatitis C treatment developments have been garnering headlines, the general public has remained largely unaware of other major liver health issues. Alcohol-associated cirrhosis is thought to account for more than one-quarter of all deaths from cirrhosis annually, and alcohol-associated cirrhosis and alcohol-associated liver cancer together cause an estimated 432,000 deaths annually [11].

Regional variations have long been noted, with North America and Europe leading other regions, but the global epidemiology of alcoholic liver disease is shaped in part by complex economic and social factors that are in a state of flux. Decreases in the price of alcohol in some countries, for example, or changes in gender norms or other social norms can lead to rapid increases in alcohol consumption, with incidence of hepatic disease increasing in turn. Developing effective public health policies to address these dynamics requires the cross-disciplinary integration of state-of-the-art knowledge from hepatologists, epidemiologists, economists, addiction researchers, and other diverse experts.

Similarly, as non-alcoholic fatty liver disease (NAFLD) becomes an increasingly prominent health threat in many parts of the world, the field of hepatitis is being called upon to address this issue through not only biomedical pathways but also through the translation of biomedical knowledge into sound evidence-based health policies. While obesity and diabetes are major risk factors for NAFLD, much work remains to be done to understand the mediating role of ethnic origin, gender and other genetic and social factors [12, 13]. The clinical management of NAFLD is not well understood either, although the available evidence suggests that simplistic efforts to change the diets and physical activity levels of people with NAFLD are unlikely to achieve major reductions in the burden of disease from this condition [14].

## Needed: a more unified response

Most troubling from my perspective is the lack of a unified strategic response to viral hepatitis and other liver diseases. How can policy-makers, researchers, health service providers, non-governmental organizations, patient groups and affected communities harmonize their efforts to address such a complex situation? What can these actors contribute to each other’s work?

Most crucially, how can we collectively ensure that the best new knowledge about disease prevention and treatment is translated as quickly as possible into national- and local-level interventions that will avert unnecessary suffering and death?

The impetus for launching *HMAP* is the recognition that no suitable forum exists for rigorously examining these questions on an ongoing basis. The established hepatology journals are certainly publishing valuable biomedical and clinical research findings. But it is imperative to also focus more squarely on health systems and the people who make up health systems—as service providers, decision-makers, patients and members of affected communities.

## HMAP’s vision

*HMAP* seeks to publish policy, public health and social science articles as well as clinical science articles from researchers who are eager to reach a wide audience. We want to expand the evidence base by addressing issues as wide-ranging as disease prevention and management, epidemiology, economics, health behavior, health service delivery, ethics, human rights, and the role of laws, policies and clinical guidelines in shaping health initiatives.

We encourage researchers working in all of these areas to highlight the policy implications of their findings—as well as the practical implications for health service providers and for people living with liver diseases or at high risk. And we encourage non-researchers to “talk back” via commentaries, roundtable discussion articles, conference events and online platforms, including the *HMAP* blog (featured in the BioMed Central “On Health” blog) [15].

In these and other ways, we greatly look forward to fostering an ongoing dialogue among the many stakeholders who are driving progress in the field of hepatology.

*HMAP* has come into being at an exciting time. The journal began accepting submissions slightly more than a year after the World Health Assembly passed its second viral hepatitis resolution in 2014, calling on governments to take more concrete actions to combat these diseases [16]. In September 2015, the first-ever World Hepatitis Summit brought together stakeholders from 84 countries—including representatives of patient groups, national governments, WHO and the pharmaceutical industry—for an unprecedented series of discussions about what the global response to viral hepatitis should look like [3]. And in May 2016, the World Health Assembly is expected to approve the World Health Organization’s first-ever global health sector strategy on viral hepatitis [1].

These developments, coupled with the dramatic advances in hepatitis C treatment, doubtlessly explain why *HMAP* has received a considerable number of submissions and pre-submission inquiries addressing hepatitis C specifically. Of course, I embrace the prospect of continuing to make progress in this area. At the same time, I recognize that the current attention to HCV offers a strategic opportunity to more comprehensively address the full constellation of biomedical and social issues relating to liver health. I am eager to see *HMAP* publish research and policy articles that help to drive forward this broader agenda, and am extremely grateful to *HMAP’s* editorial board and associate editors for helping to point the way forward.
